# Perception, knowledge and protective practices for surgical staff handling antineoplastic drugs during HIPEC and PIPAC

**DOI:** 10.1515/pp-2021-0151

**Published:** 2022-04-13

**Authors:** Hubert Benoist, Clarisse Eveno, Sarah Wilson, Nicolas Vigneron, Jean-Marc Guilloit, Rémy Morello, Nicolas Simon, Pascal Odou, Guillaume Saint-Lorant

**Affiliations:** Normandie Université, UNICAEN, UNIROUEN, ABTE, Centre de Lutte contre le Cancer F. Baclesse, Caen, France; Department of Digestive Surgery, CHU , Lille, France; CHU de Caen Normandie, Normandie Université, UNICAEN, Biostatistic and Clinical Research, Caen, France; Department of Surgery, Comprehensive Cancer Center F. Baclesse, Caen, France; Univ. Lille, CHU Lille, ULR 7365 – GRITA – Groupe de Recherche sur les Formes Injectables et les Technologies Associées, Lille, France

**Keywords:** antineoplastic drugs, HIPEC, occupational risks, PIPAC

## Abstract

**Objectives:**

Two surgical techniques used for peritoneal metastasis involve a risk of exposure to antineoplastic drugs (ADs): hyperthermic intraperitoneal chemotherapy (HIPEC) and pressurized intraperitoneal aerosol chemotherapy (PIPAC). The objective of this study was to assess the differences in perception, training, and knowledge of the risks as well as in the protection practices and occupational exposures of all worker categories.

**Methods:**

This descriptive study, led in two hospitals from two distant French regions, was performed through a face-to-face interview and assessed the perception, knowledge and handling practices of ADs by a questionnaire consisting of 52 questions.

**Results:**

Fifty-one professionals participated in this survey. A total of 29.4% (n=15) professionals were afraid to handle ADs. Very few workers have been trained on handling ADs during initial training dedicated to all caregiver (5.9%; n=3). HIPEC is considered to involve a higher risk of exposure to ADs than PIPAC (81.6% (n=31) vs. 57.9% (n=22), respectively, p=0.022, agreement 65.8%). Protective equipment is considered to be less suitable for HIPEC than for PIPAC (29% (n=11) vs. 10.5% (n=4), respectively, p=0.016, agreement 81.6%). Concerning the potential AD contamination location, the participants identified a significant difference between these two practices. During HIPEC, 15.7% (n=6) of caregivers indicated that they had negative symptoms perceived in their practice vs. 2.6% (n=1) during PIPAC.

**Conclusions:**

This study shows that perception, knowledge and protection practices are different between HIPEC and PIPAC. It also shows a difference between the worker categories. In view of the difficulties in making operating room staff available, the related training programmes must have an adapted format.

## Introduction

Peritoneal metastasis is a disseminated cancer of the peritoneum with a poor prognosis and restricted therapeutic options [1, 2]. There are two alternative techniques to systemic antineoplastic drugs (ADs) to reduce systemic side effects owing to local homogeneous penetration into the tumour tissue: hyperthermic intraperitoneal chemotherapy (HIPEC) and pressurized intraperitoneal aerosol chemotherapy (PIPAC).

The HIPEC technique consists of administering a bath of ADs into the abdominal cavity after cytoreductive surgery. The drugs are injected into the abdominal cavity at 42 ± 1 °C for a period of 30–90 min [3, 4]. The drugs used are mitomycin C for 60 min at 42 °C +/−1 °C, sometimes associated with cisplatin among others.

The PIPAC technique, developed by a German team [5], is a more recent technique. It consists of the injection of unheated ADs into the peritoneal cavity as an aerosol under pressure during laparoscopy. The therapeutic aerosol is maintained at 12 mmHg for 30 min at body temperature. The ADs used are doxorubicin and cisplatin or oxaliplatin among others.

Concerning surface contamination, no standards exist regarding the acceptable or allowable surface concentrations of ADs in the healthcare setting. Surface contamination levels for cyclophosphamide in early studies led United States Pharmacopeia to describe a 1 ng/cm^2^ level for cyclophosphamide, above which drug uptake was believed to occur [6]. For many years, the contamination of surfaces by ADs has been highlighted both in care units and in compounding units. Meijster et al. [7]. identified the different sectors potentially exposed to ADs, especially home care, nursing homes, pharmacies, laundries, waste treatment and pharmaceutical industries. In view of the risk of toxicity and contamination by ADs, healthcare workers must be particularly aware of the risks and respectful of protective practices. For nurses working in hospitals, exposure knowledge and perceived risk of harm from AD exposure were reported to be high, but the overall degree of caution exercised was low [8]. Previous studies have shown a gap between knowledge and protective practices such as using gloves [9, 10]. For staff performing HIPEC and PIPAC, perception and knowledge of the risks of handling ADs must be at the highest possible level given the data available about contamination. In the same way, the protection practices must be fully respected.

The objective of this study was to compare the differences in perception and knowledge of the risks associated with the practice of HIPEC and PIPAC, training, protection practices and occupational exposures of all staff by using questionnaires.

## Materials and methods

### Study setting

The study was led in two hospitals from two distant French regions. The first hospital has a range of the yearly number of HIPEC procedures done between 4 and 19 and a range between 6 and 11 for PIPAC procedures. The second hospital has a range of the yearly number of HIPEC procedures done between 20 and 34 and a range between 7 and 35 for PIPAC procedures. The caregivers were selected through recruitment among the operating room staff and signed a letter of informed consent.

### Questionnaire design

The questionnaire was approved by the local committee for the protection of the persons concerned (number A16-D49-VOL.30). Draft versions of the questionnaires were pretested on a small group of healthcare workers who did not participate in the research project. The questionnaires included 52 questions and were divided into three major sections, namely, general questions, questions on HIPEC and questions on PIPAC, as presented in Appendix 1. The last two sections included questions on perception, knowledge, training on protection practices and occupational exposure. The finalized questionnaires first included participant characteristics such as gender, age and work experience. The first question which was general and related to risk perception was adapted from a survey led by Hon et al. [10] and had response options of “agree/disagree/don’t know”. The others questions, specific to risk perception during HIPEC and PIPEC had response options of very low to very important. The questions related to knowledge had response options of “agree/somewhat agree/somewhat disagree/disagree”, while the questions regarding training and knowledge of the existing procedures had more often a response options of “true/false/don’t know” adapted to the question. The questions related to risk handling practice and exposure practice had response options of “true/false”, while questions regarding personal protective equipment had response options of “always/sometimes/never”.

### Data collection

The questionnaires were administered during individual 20-min interviews led by two investigators, non-members of the surgical teams, who developed the questionnaire and consulted each other when in doubt. Interviews were not recorded so as not to create stress that could impact the answers to the questions. Carrying out the interview of caregiver during a break time and not on activity time. The survey was carried out between September 2019 and March 2020. Once all the questionnaires were collected, they were coded and input into EpiData version 3.1 software (EpiData association, Odense). All data were input by the same person other than the two investigators and were double-checked by an epidemiologist.

### Statistical analysis

General characteristics were primarily described for all the participants, then profession characteristics. The participants who performed both HIPEC and PIPAC were retained for paired analysis comparing their risk perception, knowledge and practices between the two procedures. The answers of these participants were then compared according to their profession. Qualitative variables were described with their effectives and percentages and compared using the chi-squared test or Fisher’s exact test if at least one of the expected values was lower than 5. Paired qualitative data were described with their effectives and percentages and compared using the McNemar chi-square test, and their concordance was assessed by the agreement rate and Cohen’s kappa. A kappa value ≤ 0.20 represents poor/slight agreement, from 0.21-0.40 indicates fair agreement, from 0.41-0.60 represents moderate agreement, from 0.61-0.80 means good agreement and ≥ 0.81 indicates excellent agreement. Quantitative variables were described by their mean, standard deviation and minimum and maximum. The means were compared using Student’s t-test or paired Student’s t-test for paired data. A p-value lower than 0.05 was considered significant. Data management and statistical analysis were performed using IBM SPSS Statistics for Windows, Version 23.0. Armonk, NY: IBM Corp.

## Results

Fifty-one healthcare professionals participated in this study out of the 54 solicited (response rate: 94.4%) (Table 1A). There were 16 caregivers from one hospital and 35 from the other, representing 7 different occupational categories grouped into three major categories working in the operating room: medical staff (n=10), nursing staff (n=20) and auxiliary caregivers (n=21). The gender ratio M/F was 0.7. The majority of the health professionals surveyed worked in operating rooms during their hospital careers (Table 1A). The average length of employment was shorter for medical staff than for nursing staff due to the presence of assistants and residents in the medical staff. The average length of employment for auxiliary caregivers was fairly short, given their high turnover. Generally, outside the context of HIPEC and PIPAC, 29.4% (n=15) of healthcare professionals were afraid to handle ADs (Table 1B). Concerning fear, there was a significant difference among occupational workers (p=0.012), particularly in auxiliary caregivers. For the other questions, no significant differences were observed. We can notice that 15.7% of participants (n=8) thought that the toxicity risk level is the same for all ADs and 74.5% (n=38) thought that the safety measures currently in place sufficiently reduce the risk of contamination. For nursing staff and auxiliary staff, barely half of the respondents were confident that they can handle all situations with a potential risk of exposure, at 35% (n=7) and 52.0% (n=11), respectively. It is worth mentioning that very few staff have been trained on handling ADs during initial training dedicated to all occupational staff (5.9%, n=3). In addition, only 29.4% (n=15) were trained to handle ADs in their current place of work, with a mean period of 1.8 years since the last ongoing training program. In this survey, among the participants performing the two techniques (n=38), 76.3% (n=29) wished to be trained in the proper handling procedures for HIPEC and 71.0% (n=27) for PIPAC. Among respondents wanted to be trained on AD handling during HIPEC (n=29), 44.8% (n=13) would like to follow training in an academic lecture format and 20.7% (n=6) would like to follow distance e-learning training. For PIPAC, 51.8% (n=14) of respondents wanted to be trained (n=27) wished to follow a training course in an academic lecture format, while only 18.5% (n=5) wanted a distance e-learning course.

**Table 1A:** Participants’ characteristics.

		Overall	Medical staff	Nursing staff	Auxiliary caregivers
Effectives, n (%)		51 (100)	10 (19.6)	20 (39.2)	21 (41.2)
Gender	Female, n (%)	30 (58.8)	3 (30)	13 (65)	14 (66.7)
	Male, n (%)	21 (41.2)	7 (70)	7 (35)	7 (33.3)
Job tenure in the hospital, mean ± SD (range), months		137.6 ± 115.1 (2–467)	57.3 ± 74.5 (2–251)	160.4 ± 98.4 (7–419)	154.2 ± 131.9 (36–467)
Job tenure in operating rooms, mean ± SD (range), months		94.4 ± 72.1 (7–275)	75.7 ± 76.3 (12–251)	111.1 ± 79.9 (7–275)	87.5 ± 61.7 (24–251)
Mean job tenure in HIPEC, mean ± SD (range), months		43.8 ± 44.4 (1–168)	27.3 ± 44.5 (1–144)	68.1 ± 51.6 (7–168)	28.5 ± 23.2 (12–120)
Mean job tenure in PIPAC, mean ± SD (range), months		12.1 ± 11.1 (<1–36)	12.3 ± 10.3 (1–24)	15.8 ± 13.2 (<1–36)	7.8 ± 7.4 (4–24)
Age, mean ± SD (range), years		40.0 ± 9.3 (24–58)	35.3 ± 8.3 (27–56)	41.3 ± 7.4 (30–58)	41.1 ± 11.0 (24–57)

**Table 1B:** Participants’ characteristics.

Questions		Overall (n=51)	Medical staff (n=10)	Nursing staff (n=20)	Auxiliary caregivers (n=21)	p-Value
Fear of handling AD [Q3]	Agree	15 (29.4)	1	3	11	**0.012**
	Disagree	36 (70.6)	9	17	10	
The toxicity risk level is the same for all AD [Q4]	Agree	8 (15.7)	2	4	2	0.598
	Disagree + Don’t know	43 (84.3)	8	16	19	
The safety measures currently in place sufficiently reduce the risk of contamination [Q5]	Agree	38 (74.5)	8	15	15	1.000
	Disagree + Don’t know	13 (25.5)	2	5	6	
I am confident that I can handle all situations with a potential risk of exposure to AD [Q6]	Agree	25 (49.0)	7	7	11	0.187
	Disagree + Don’t know	26 (51.0)	3	13	10	
Initial training on the handling of AD (at nursing school, medical school, etc.) [Q7]	Yes	3 (5.9)	1	2	0	0.297
	No	48 (94.1)	9	18	21	

The bold values indicate a significant difference p < 0.05.

Comparing the differences in perceived risks associated with the practice of HIPEC and PIPAC (Table 2A), HIPEC is considered to have a higher risk of exposure to ADs than PIPAC (81.6% (n=31) vs. 57.9% (n=22), respectively, p=0.022, agreement 65.8%). In connection with this question, protective equipment is considered to be less suitable for HIPEC than for PIPAC (29 vs. 10.5%, respectively, p=0.016, agreement 81.6%). The risk of exposure is perceived as not being very low, especially for auxiliary caregivers in HIPEC, and is statistically significant among occupational categories for both HIPEC (p=0.018) and PIPAC (p=0.004) (Table 2B).

**Table 2A:** Perception questions.

Questions		Procedure performed			Concordance		
		HIPEC (n=38)	PIPAC (n=38)	p-Value	Kappa	p-Value	Agreement
Risk exposure assessment [Q13 Q31]	Very low	7	16	**0.022**	0.240	0.108	65.8
	≥ Low	31	22				
Suitable protective equipment [Q14 Q32]	Agree	27	34	**0.016**	0.448	0.005	81.6
	Disagree + Don’t know	11	4				

The bold values indicate a significant difference p < 0.05.

**Table 2B:** Perception questions by worker categories.

Questions		Worker category n=38			
		Medical staff (n=6)	Nursing staff (n=17)	Auxiliary caregivers (n=15)	p-Value
Risk exposure assessment for HIPEC [Q13]	Very low	3	4	0	**0.018**
	≥ Low	3	13	15	
Risk exposure assessment for PIPAC [Q31]	Very low	5	9	2	**0.004**
	≥ Low	1	8	13	
Is protective equipment suitable for HIPEC ? [Q14]	Agree	6	12	9	0.247
	Disagree + Don’t know	0	5	6	
Is protective equipment suitable for PIPAC ? [Q32]	Agree	6	16	12	0.362
	Disagree + Don’t know	0	1	3	

The bold values indicate a significant difference p < 0.05.

A significant difference can be observed between HIPEC and PIPAC in the ongoing training on the handling of ADs (p=0.039) (Table 3A) as well as between workers’ categories in the PIPAC technique (p=0.010) (Table 3B). As complementary data to the Tables, 41.2% (n=21) of workers were not made aware of the correct procedure in case of accidental exposure to ADs, with a significant difference between worker categories (p=0.006): medical staff, n=5 (50%), nursing staff, n=3 (15%), auxiliary caregivers, n=13 (61.9%). They also did not know about the elimination procedure for ADs (p=0.037), particularly among nursing staff. Concerning the main potential routes of contamination perceived by the participants in the practice of HIPEC and PIPAC, significant differences were observed: workers identified the cutaneous route (92.1% (n=35) vs. 63.2% (n=24), p=0.003, agreement 65.8%) as well as the ocular route (81.6% (n=31) vs. 50.0% (n=19), p=0.002, agreement 63.2%) and the injectable route of contamination (57.9% (n=22) vs. 36.8% (n=14), p=0.008, agreement 78.9%) (Table 3A). More precisely, the non-medical category did not identify the cutaneous route during HIPEC practice (p=0.027) (Table 3B). Concerning the potential AD-contaminated location, the participants identified a significant difference between these two practices, namely, regarding contamination of the operating table (86.8% for HIPEC (n=33) vs. 65.8% for PIPAC (n=25), p=0.039, agreement 68.4%) and regarding contamination of the floor (94.7% for HIPEC (n=36) vs. 76.3% for PIPAC (n=29), p=0.016, agreement 81.6%) (Table 3A). More precisely, for HIPEC, the nursing staff and auxiliary caregivers did not identify any potential floor contamination, in contrast to the medical staff (p=0.022) (Table 3B). We can notice that for injectable route, there was a significant difference between worker categories (p=0.0001). For the other locations, no significant differences were observed for the different participants.

**Table 3A:** Knowledge questions.

Questions			Procedure performed (n=38)			Concordance		
			HIPEC	PIPAC	p-Value	Kappa	p-Value	Agreement
Ongoing training specific to this technique [Q15 Q33]		Yes	12	5	**0.039**	0.350	0.026	76.3
		No	26	33				
Potential contamination by:	Injectable route [Q19a Q37g]	Agree	22	14	**0.008**	0.596	<0.001	78.9
		Disagree	16	24				
	Cutaneous route [Q19b Q37h]	Agree	35	24	**0.003**	0.121	0.548	65.8
		Disagree	3	14				
	Inhaled route [Q19c Q37i]	Agree	22	25	0.607	0.169	0.315	60.5
		Disagree	16	13				
	Ocular route [Q19d Q37j]	Agree	31	19	**0.002**	0.263	0.086	63.2
		Disagree	7	19				
	Oral route [Q19e Q37k]	Agree	21	15	0.109	0.485	0.002	73.7
		Disagree	17	23				
	Infusion bags [Q20a Q38a]	Agree	23	25	0.625	0.774	<0.001	89.5
		Disagree	15	13				
	Operating table [Q20d Q38d]	Agree	33	25	**0.039**	0.177	0.309	68.4
		Disagree	5	13				
	Trolley surfaces [Q20e Q38e]	Agree	24	16	0.057	0.293	0.085	63.2
		Disagree	14	22				
	Screen surfaces [Q20f Q38f]	Agree	21	14	0.143	0.129	0.506	55.3
		Disagree	17	24				
	Ventilator [Q20g Q38g]	Agree	18	14	0.424	0.253	0.178	63.2
		Disagree	20	24				
	Operating room telephone [Q20h Q38h]	Agree	22	19	0.508	0.526	0.002	76.3
		Disagree	16	19				
	Computer keyboard [Q20i Q38i]	Agree	21	18	0.508	0.529	0.002	76.3
		Disagree	17	20				
	Door detector [Q20j Q38j]	Agree	16	15	1.000	0.401	0.019	71.1
		Disagree	22	23				
	Floor [Q20k Q38k]	Agree	36	29	**0.016**	0.304	0.055	81.6
		Disagree	2	9				
	Walls [Q20l Q38l]	Agree	23	17	0.146	0.382	0.020	68.4
		Disagree	15	21				
	Smoke evacuation system [Q20m Q38m]	Agree	23	22	1.000	0.510	0.003	76.3
		Disagree	15	16				
	Other [Q20n Q38o]	Yes	5	1	0.125	0.303	0.135	89.5
		No	33	37				

The bold values indicate a significant difference p < 0.05.

**Table 3B:** Knowledge questions by worker categories.

Questions			Worker category (n=38)			
			Medical staff (n=6)	Nursing staff (n=17)	Auxiliary caregivers (n=15)	p-Value
Ongoing training specific to this technique for HIPEC [Q15]		Yes	1	2	9	**0.010**
		No	5	15	6	
Ongoing training specific to this technique for PIPAC [Q33]		Yes	1	2	2	1.000
		No	5	15	13	
HIPEC potential contamination by:	Injectable route [Q19a]	Agree	3	7	12	0.084
		Disagree	3	10	3	
	Cutaneous route [Q19b]	Agree	4	17	14	**0.027**
		Disagree	2	0	1	
	Ocular route [Q19d]	Agree	4	15	12	0.478
		Disagree	2	2	3	
	Operating table [Q20d]	Agree	4	16	13	0.216
		Disagree	2	1	2	
	Floor [Q20k]	Agree	4	17	15	**0.022**
		Disagree	2	0	0	
PIPAC potential contamination by:	Injectable route [Q37g]	Agree	1	2	11	**0.001**
		Disagree	5	15	4	
	Cutaneous route [Q37h]	Agree	2	10	12	0.129
		Disagree	4	7	3	
	Ocular route [Q37j]	Agree	2	7	10	0.320
		Disagree	4	10	5	
	Operating table [Q38d]	Agree	2	11	12	0.146
		Disagree	4	6	3	
	Floor [Q38k]	Agree	3	12	14	0.075
		Disagree	3	5	1	

The bold values indicate a significant difference p < 0.05.

Concerning personal protective equipment (PPE), a tendency to wear specific gloves was observed, which was more frequent in HIPEC (97.3% (n=37) for HIPEC vs. 84.0% for PIPAC (n=32), p=0.063, agreement 86.8%) (Table 4A). The systematic wearing of specific masks was similar in both HIPEC and PIPAC practices; protective goggles were not systematically worn by workers for either surgical technique (65.7% for HIPEC (n=25) vs. 52.6% for PIPAC (n=20), respectively). Concerning general participants’ characteristics (n=51), 54.9% (n=28) of the respondents indicated that they wear glasses. However, the wearing of a gown varied significantly between these surgical practices (86.8% for HIPEC (n=33) vs. 63.2% for PIPAC (n=24), p=0.004, agreement 76.3%), with no difference between worker categories (Table 4B). Following the evaluation of protective practices, the exposure to antineoplastic drugs and the clinical signs felt during HIPEC and PIPAC were also investigated. During HIPEC, 6 healthcare professionals (15.7%) indicated that they had negative symptoms perceived in their current practice vs. one caregiver (2.6%) during PIPAC (Table 4A). These professionals had headaches, and one indicated that he also had skin signs. No surgeons who were closest to ADs reported any clinical signs. Five other professionals reported accidental exposure to ADs during these surgical techniques and did not experience any clinical signs during these accidental exposures.

**Table 4A:** Practice questions.

Questions		Procedure performed (n=38)			Concordance		
		HIPEC	PIPAC	p-Value	Kappa	p-Value	Agreement
Bio-decontamination procedure knowledge [Q21 Q39]	Yes	31	25	0.109	0.343	0.037	73.7
	No + Don’t know	7	13				
If yes, can you explain it?	Yes	15	15	1.000	0.850	<0.001	92.6
	No	12	12				
Wearing gloves [Q22 Q40]	Always	37	32	0.063	0.252	0.160	86.8
	Not always	1	6				
Wearing a mask [Q23 Q41]	Always	36	35	1.000	0.360	0.158	92.1
	Not always	2	3				
Wearing protective goggles [Q24 Q42]	Always	25	20	0.125	0.625	<0.001	81.6
	Not always	13	18				
Wearing a gown [Q25 Q43]	Always	33	24	**0.004**	0.412	0.004	76.3
	Not always	5	14				
Mean annual number of interventions [Q26 Q44]	<10	13	13	1.000	0.766	<0.001	89.5
	≥10	25	25				
Bio-decontamination activity [Q27 Q45]	Yes	21	17	0.125	0.792	<0.001	89.5
	No	17	21				
If yes, frequency of bio-decontamination	Each patient	14	15	1.000	–	–	94.1
	Once a day	3	2				
Use of a proper technique for bio-decontamination	Yes	14	14	1.000	1.000	0.065	100.0
	No	1	1				
Negative symptoms perceived when not exposed [Q28 Q46]	Yes	6	1	0.125	−0.047	1.000	81.6
	No	32	37				
Ever exposed to AD [Q29 Q47]	Yes	2	3	1.000	−0.067	1.000	86.8
	No	36	35				

The bold values indicate a significant difference p < 0.05.

**Table 4B:** Practice questions by worker categories.

Questions		Worker category n=38			
		Medical staff (n=6)	Nursing staff (n=17)	Auxiliary caregivers (n=15)	p-Value
Wearing a gown [Q25]	Always	4	16	13	0.218
	Not always	2	1	2	
Wearing a gown [Q43]	Always	4	8	12	0.159
	Not always	2	9	3	

## Discussion

This study aimed at showing if there were differences in perception and knowledge of the risks associated with the practice of HIPEC and PIPAC, training, protection practices and occupational exposures of all staff by using questionnaires. This multi-centre study showed a difference in safety perception and knowledge of the risk of exposure to ADs between HIPEC and PIPAC as well as between occupational categories, especially in the practice of HIPEC, which was considered by the participants to be riskier than PIPAC.

This study was conducted in two hospitals, a large academic hospital with versatility of activities and a smaller cancer-specific hospital corresponding to the two types of hospitals performing these procedures. For this study, there were few participants which can be explained by a specialized activity. However, the rate of participation in this questionnaire was very high (94.4%), i.e., almost all the operating room staff in the two centres. The questionnaires were conducted face-to-face and not by sending by post a self-administered questionnaire. A face-to-face questionnaire allows avoiding missing data. Such an interview can be stressful for the caregivers, but in the context of this study, the interviews were conducted during the same period by two investigators non-workers in the operating rooms and centres, during a break time and not during the activity time.

Protective equipment is considered to be less suitable for HIPEC than for PIPAC. Al Hosni et al. [11] assessed the awareness of the non-medical operating theatre staff concerning the risk of contamination. The authors found similar results, with half of the participants who believed that there was a moderate risk of exposure to ADs with HIPEC, while regarding PIPAC, this risk was estimated as weak for 59.0% of the respondents. The risk of exposure was perceived as being not very low in both practices, especially for auxiliary caregivers, and was statistically significant between the occupational categories for both HIPEC and PIPAC. Clerc et al. [12] also showed that more than other worker categories, operating room cleaning staff perceived the main risks of AD exposure in HIPEC but not in PIPAC. The safety perceived for PIPAC could have various explanations. The amount and volume of drug used in PIPAC is up to 10-fold lower than that in HIPEC. During the PIPAC procedure, the chemotherapy liquid is aerosolized, unlike in the HIPEC procedure, where the ADs remains liquid. In open-abdomen HIPEC, called the coliseum technique, the chemotherapy drug is released in the open air, unlike in the closed abdomen technique. PIPAC is administered during laparoscopy, and the patient is placed under a plastic cover to avoid the risk of contamination in case of leakage. During the whole process of injection of ADs and the residence time during PIPAC, the operating staff is outside the operating room.

Knowledge of risk exposure was less perceived in PIPAC, particularly concerning perception of the main potential routes of contamination such as the cutaneous route or the ocular route. Therefore, staffs are potentially exposed to aerosol contamination such as in the PIPAC technique or cutaneous contamination. The results are particularly important for the risk of contamination as at least 41.2% of workers had not been made aware of the proper procedure in case of accidental exposure to ADs, with a significant difference between the categories of workers. The workers, particularly nursing staff, also did not know about the elimination procedure of ADs.

A review by Kyriazanos et al. [13] concerning the safety of the operating staff during the administration of HIPEC showed that this procedure can be safely performed with regard to occupational exposure of healthcare staff. Indeed, compliance with specific protective measures is a key factor in minimizing AD exposure. Ametsbichler et al. [14] showed that AD contamination on various operating room surfaces is widespread and can lead to a distribution of AD residues and that the air contamination was very low. The risk of exposure for PIPAC is very low if adequate safety and cleaning standards are respected. Indeed, this potential AD contamination appears to originate from contact with contaminated surfaces. Many studies in the literature have shown that ADs are still regularly detected on surfaces in healthcare centres despite the specific decontamination protocols implemented [15, 16]. In our study, workers underestimated the environmental contamination, particularly the nursing staff and auxiliary caregivers who did not identify the operating table as potentially contaminated in PIPAC or floor contamination in HIPEC, unlike medical staff. In HIPEC, platinum surface contamination was found on the operating table (1.9–15 pg/cm^2^), on the floor under the operating table both before HIPEC (3.7–82 pg/cm^2^) and after HIPEC (970–19,458 pg/cm^2^) and up to 5 m on the floor (max: 36 pg/cm^2^), on the surgeon’s shoes (6.5–134 pg/cm^2^) and on the infusion bags (12–88 pg/cm^2^) [17]. Platinum surface contamination was also found on HIPEC devices, especially on the regulation knob, with a median of approximately 10 pg/cm^2^ [18]. For PIPAC, surface contamination by platinum was found in the injector (median: 2.82 pg/cm^2^) and trocars (median: 9.42 pg/cm^2^) and to a lesser extent on the floor (median: 0.06 pg/cm^2^) [14]. These results were confirmed more precisely in another study, which was also published in 2018, with platinum contamination of up to 574 ng/wipe for the neck injector syringe holder after cleaning was done [19]. Noted that in a recent French study, no surface contamination with platinum compounds were detected during PIPAC and 25% of urine samples in the exposed caregivers group were contaminated with no statistical difference observed in samples collected before and after PIPAC [20].

The lack of knowledge of the contamination risk could be explained by the non-systematic wearing of specific PPE, such as protective goggles that were not systematically worn by workers for both surgical techniques. The wearing of a gown was significantly different between these surgical practices. In one of the largest surveys on health care workers in the United States performed in care units, Boiano et al. [21] found that safe handling practices were not always respected by nurses who administer ADs, even though guidelines have existed for some time. Recently, in the context of peritoneal metastasis, Al Hosni et al. [11] showed that chemotherapy was identified as an occupational risk for all caregivers and explained that the partial wearing of PPE was due to the presence of slips and lapses and by a lack of comfort.

However, professionals have reported accidental exposure to ADs during these surgical techniques. During HIPEC, healthcare professionals indicated that they had negative symptoms during their current practice, with one caregiver reporting headaches and skin signs during PIPAC. It is, however, a difficult argument to prove that the headaches reported by professionals during HIPEC are linked to exposure to ADs. Indeed, HIPEC is a long surgery. Perception of environmental safety among operating room staff participating in HIPEC was previously investigated by Ortega-Deballon et al. [22]. They observed that operating room staff reported having experienced noxious symptoms during open HIPEC procedures (55%) and that this incidence of symptoms fell to 17% after the introduction of the ‘‘semi-open’’ technique. The symptoms felt in the context of AD handling were previously investigated in care units [23], [24], [25], [26]. Nevertheless, these accidental exposures should not be taken lightly, especially since our study shows a lack of knowledge of the exposure risk as well as defects in handling practices.

This study highlights a demand for training in the handling of ADs and protective measures. The participants wished to be trained in the proper procedures of AD handling. However, the response to questions regarding training dates is limited to the participants’ recollection and not to the real training dates. Al Hosni et al. [11] also highlighted the need for training in the context of HIPEC and PIPAC. Among those who would like to be trained, a majority of respondents would like to follow a training course in an academic lecture format. During the data collection and analysis of the results of the questionnaires, no causes for the choice of e-learning or academic lecture format were found for the minority of respondents who want to follow an e-learning course. Only 29.4% were trained to handle ADs in their current place of work, with a significant difference depending on the worker category in the PIPAC technique. The impact on practices of in-service training was previously studied in care units [9, 23, 27]. The different results of this study as well as the disparity between the worker categories show a need for a training programme that would be suited to the various work categories because the needs vary from one category to another.

## Conclusions

This study shows that perception, knowledge and protection practices vary between HIPEC and PIPAC. It also shows differences between these elements depending on the occupational categories. These results show the importance of implementing training programmes that tackle the contamination risks by ADs and take occupational categories into account. In view of the difficulties in making operating room staff available, the training programmes must have an adapted format using, for instance, simulation or e-learning. A better knowledge of the risks makes it easier to protect oneself properly.

## Supplementary Material

Supplementary Material

Supplementary Material
