# Supplementary material for: Perception, knowledge and protective practices for surgical staff handling antineoplastic drugs during HIPEC and PIPAC
Source: Pleura Peritoneum. 2022 Apr 13;7(2):77–86. doi: 10.1515/pp-2021-0151 (PMC9166181; doi:10.1515/pp-2021-0151)
Supplement: Supplementary file 1 — Supplementary Material [file j_pp-2021-0151_suppl_001.docx]

**Questionnaire général « étude CONTACT » Bloc CHIP/PIPAC**

**Ecrire en lettres MAJUSCULES, Cochez les cases, Utilisez un crayon noir**

**Nom :**

**Prénom :**

**Sexe : □ Féminin □ Masculin**

**Date de naissance : |_|_|/|_|_|/|_|_|_|_|**

**Fonction : □ IBODE □ IADE □ IDE □ Médecin □ Cadre □ AS □ ASH**

**□ Interne □ Etudiant IBODE □ Etudiant IADE □ AS/ASH en formation**

**□ Autre :**

**Etes-vous rattaché spécifiquement aux BLOCs ? □** Oui  □ Non

**Si non, à quel taux horaire êtes-vous rattaché aux BLOCs (pourcentage) ?**

**à quel autre unité êtes-vous rattachée ?**

**Ancienneté dans l’établissement ?**  Valeur : en □ semaines □ mois □ années

**Ancienneté aux BLOCs  (durée continue) ?** Valeur : en □ semaines □ mois □ années

**Partie 1 : Questions générales**

**1- Jugez-vous votre risque d’exposition général aux anticancéreux très faible ?**

□ D’accord □ Pas d’accord □ je ne sais pas

**2- Entre la CHIP et la PIPAC, laquelle jugez-vous la moins à risque d’exposition aux anticancéreux ?**

□ CHIP □ PIPAC

**3- Avez-vous peur de manipuler des anticancéreux ?**

□ D’accord □ Pas d’accord □ je ne sais pas

**4- Pensez-vous que les anticancéreux soient tous au même niveau de risque de toxicité ?**

□ D’accord □ Pas d’accord □ je ne sais pas

**5- Pensez-vous que les mesures de sécurité mises en place actuellement permettent de réduire suffisamment le risque de contamination ?**

□ D’accord □ Pas d’accord □ je ne sais pas

**6- Je suis confiant(e) dans le fait que je puisse gérer toutes les situations où il y a une exposition potentielle aux anticancéreux ?**

□ D’accord □ Pas d’accord □ je ne sais pas

**7- Avez-vous été formé(e) spécifiquement à la manipulation des anticancéreux dans votre formation initiale (IFSI, internat) ?** □ Oui □ Non

**8 - Avez-vous été formé(e) spécifiquement à la manipulation des anticancéreux dans votre formation continue (dans votre établissement actuel) ?** □ Oui □ Non

**Si oui, de quand date votre dernière formation sur ce sujet ?**  |_|_|/|_|_|/|_|_|_|_|

**durée de la formation réalisée ?** Valeur : en □ minutes □ heures □ jours

**9- Existe-t-il une procédure « conduite en cas d’exposition accidentelle aux anticancéreux »?**

**□** Oui  □ Non □ je ne sais pas

**Si oui, êtes-vous en capacité de l’expliquer ?** □ Oui □ Non

**10- Que faites-vous ou feriez-vous en cas d’exposition à des cytostatiques ?**

Utilisation de SHA **□** Oui  □ Non Lavage à l’eau **□** Oui  □ Non

Utilisation de savon **□** Oui  □ Non Rien **□** Oui  □ Non

Autres ?  □ Non □ Oui, précisez :

**11- Connaissez-vous la procédure d’élimination des déchets anticancéreux ?** □ Oui □ Non

**Partie 2 : Questions sur la CHIP**

**12 - Travaillez-vous en bloc de CHIP ?** □ Oui □ Non

**Si non passez à la partie 3.**

**Si oui, ancienneté dans l’activité de CHIP ?** Valeur : en □ semaines □ mois □ années

**13**- **Comment jugez-vous votre risque d’exposition aux anticancéreux en CHIP ?**

□ Très faible □ Faible □ Importante □ Très importante

**14- Pensez-vous que les équipements de protection individuelle fournis soient adaptés (ergonomie/protection) à la manipulation des anticancéreux en CHIP ?**

□ D’accord □ Pas d’accord □ je ne sais pas

**Si pas d’accord à l’un, lesquels ?**

- Casaque(s) **□** oui □ non - Masque **□** oui □ non - Lunettes de protection **□** oui □ non

- Gants **□** oui □ non - Surchaussures □ oui □ non

- Autres ? □ non **□** oui, précisez :

**15- Avez-vous été formé(e) spécifiquement à la manipulation des anticancéreux en CHIP dans votre formation continue (dans votre établissement actuel) ?** □ Oui □ Non

**Si oui, de quand date votre dernière formation sur ce sujet ?**  |_|_|/|_|_|/|_|_|_|_|

**durée de la formation réalisée ?** Valeur : en □ minutes □ heures □ jours

**16- Souhaiteriez-vous être sensibilisé(e) aux bonnes pratiques de manipulation des anticancéreux (poster, livret information) en CHIP ?** □ Oui □ Non

**17- Souhaiteriez-vous être informé(e) aux bonnes pratiques de manipulation des anticancéreux (staff dans le service) en CHIP ?** □ Oui □ Non

**18- Souhaiteriez-vous être formé(e) aux bonnes pratiques de manipulation des anticancéreux en CHIP ?**  □ Oui □ Non

**Si oui, durée : □** 1h □ ½ journée □ ou plus

**format : □** E-learning □ Cours magistraux □ Les deux □ Aucun

**19- Selon vous quelles sont les voies de contamination possibles par les anticancéreux en CHIP?**

1. Voie injectable (piqures) :

*□ D’accord □ plutôt d’accord □ plutôt pas d’accord □ pas d’accord*

1. Voie cutanée :

*□ D’accord □ plutôt d’accord □ plutôt pas d’accord □ pas d’accord*

1. Voie inhalée :

*□ D’accord □ plutôt d’accord □ plutôt pas d’accord □ pas d’accord*

1. Voie oculaire :

*□ D’accord □ plutôt d’accord □ plutôt pas d’accord □ pas d’accord*

1. Voie orale :

*□ D’accord □ plutôt d’accord □ plutôt pas d’accord □ pas d’accord*

1. Autres ?

*□ non □ oui, précisez :*

**20- Selon vous, où peut-on retrouver des anticancéreux au sein du bloc en cas de CHIP ?**

1. Surface de la poche d’anticancéreux:

*□ D’accord □ plutôt d’accord □ plutôt pas d’accord □ pas d’accord*

1. Surface de la pompe de CHIP:

*□ D’accord □ plutôt d’accord □ plutôt pas d’accord □ pas d’accord*

1. Tubulure de CHIP:

*□ D’accord □ plutôt d’accord □ plutôt pas d’accord □ pas d’accord*

1. Table d’opération :

*□ D’accord □ plutôt d’accord □ plutôt pas d’accord □ pas d’accord*

1. Surface des chariots:

*□ D’accord □ plutôt d’accord □ plutôt pas d’accord □ pas d’accord*

1. Surface des moniteurs:

*□ D’accord □ plutôt d’accord □ plutôt pas d’accord □ pas d’accord*

1. Le respirateur:

*□ D’accord □ plutôt d’accord □ plutôt pas d’accord □ pas d’accord*

1. Téléphone du bloc:

*□ D’accord □ plutôt d’accord □ plutôt pas d’accord □ pas d’accord*

1. Clavier d’ordinateur :

*□ D’accord □ plutôt d’accord □ plutôt pas d’accord □ pas d’accord*

1. Détecteur d’ouverture de porte :

*□ D’accord □ plutôt d’accord □ plutôt pas d’accord □ pas d’accord*

1. Sol :

*□ D’accord □ plutôt d’accord □ plutôt pas d’accord □ pas d’accord*

1. Mur:

*□ D’accord □ plutôt d’accord □ plutôt pas d’accord □ pas d’accord*

1. Aspirateur fumée:

*□ D’accord □ plutôt d’accord □ plutôt pas d’accord □ pas d’accord*

1. Autres ? :

*□ Non □ Oui, précisez :*

**21- Existe-t-il une procédure spécifique de bionettoyage du bloc en cas de CHIP ?**

**□** Oui  □ Non □ je ne sais pas

**Si oui, êtes-vous en capacité de l’expliquer ?** □ Oui □ Non

**22- Portez-vous des gants en CHIP ?**

□ Toujours □ parfois □ jamais

**23- Portez-vous un masque en CHIP?**

□ Toujours □ parfois □ jamais

***Si toujours ou parfois,* quel est le type de ce masque ?**  □ FFP1 □ FFP2 □ FFP3

Autres ? : □ Non □ Oui, précisez :

**24- Portez-vous des lunettes de protection en CHIP ?**

□ Toujours □ parfois □ jamais

**25- Portez-vous une casaque en CHIP ?**

□ Toujours □ parfois □ jamais

***Si toujours ou parfois,***  **précisez l’équipement :**

**26- En moyenne, combien de CHIPs réalisez-vous par an ?**

□ 0 □ 1-4 □ 5-9 □ 10-14 □ 15-19 □ 20+

**27- Biodécontaminez-vous personnellement les surfaces exposés ? □** Oui  □ Non

**Si oui, a quelle fréquence, biodécontaminez-vous les surfaces exposées ?**

□ 1 fois par changement de patient □ 1 fois par jour

**comment ?**

- Utilisation lingettes (type stericid) **□** Oui  □ Non
- Utilisation de SURFANIOS ou equivalent  **□** Oui  □ Non
- Changez-vous de lingettes à chaque changement de surface ? □ Oui □ Non
- Autre  **□** Non  □ Oui, précisez :

**28- Avez-vous déjà ressenti des troubles (type céphalées) en bloc de CHIP** **hors exposition accidentelle ?** **□** Oui  □ Non

**29- Avez-vous été exposé(e) à des anticancéreux de façon inhabituelle?** **□** Oui  □ Non

- **Si non, passez à la partie 3**

**Si oui, à quelle fréquence :**

□ moins d’1fois par an □ 1 fois par an □ une fois par mois □ une fois par semaine □ jamais

**Par quel biais :**

- « débordements » **□** Oui  □ Non
- Déchirures des gants **□** Oui  □ Non
- Au moment de la connexion de la poche à la pompe (CHIP) **□** Oui  □ Non
- Exposition lors de l’élimination DASRI **□** Oui  □ Non
- Autre ?  **□** Non  □ Oui, précisez :

**Suite à ces expositions, avez-vous ressenti des troubles** : **□** Oui  □ Non

- **Si non, passez à la partie 3**

**Qu’avez-vous ressenti ?**

- Signes digestifs **□** Oui  □ Non
- Signes cutanés **□** Oui  □ Non

**Si oui,** - brûlures **□** Oui  □ Non - picotements **□** Oui  □ Non

- Signes neurologiques **□** Oui  □ Non

**Si oui,** - céphalées **□** Oui  □ Non - vertiges **□** Oui  □ Non

- Réactions allergiques **□** Oui  □ Non
- Signes oculaires **□** Oui  □ Non
- Autres ?  **□** Non  □ Oui, précisez :

**De façon générale, si présence d’un trouble quel qu’il soit durée moyenne des symptômes:**

Valeur : en □ minutes □ heures □ jours

**Partie 3 : Questions sur la PIPAC**

**30 - Travaillez-vous en bloc de PIPAC?** □ Oui □ Non

**Si non passez à la partie 4.**

**Si oui, ancienneté dans l’activité de PIPAC?** Valeur : en □ semaines □ mois □ années

**31**- **Comment jugez-vous votre risque d’exposition aux anticancéreux en PIPAC?**

□ Très faible □ Faible □ Importante □ Très importante

**32- Pensez-vous que les équipements de protection individuelle fournis soient adaptés (ergonomie/protection) à la manipulation des anticancéreux en PIPAC ?**

□ D’accord □ Pas d’accord □ je ne sais pas

**Si pas d’accord à l’un, lesquels ?**

- Casaque(s) **□** oui □ non - Masque **□** oui □ non - Lunettes de protection **□** oui □ non

- Gants **□** oui □ non - Surchaussures □ oui □ non

- Autres ? □ non **□** oui, précisez :

**33- Avez-vous été formé(e) spécifiquement à la manipulation des anticancéreux en PIPAC dans votre formation continue (dans votre établissement actuel) ?** □ Oui □ Non

**Si oui, de quand date votre dernière formation sur ce sujet ?**  |_|_|/|_|_|/|_|_|_|_|

**durée de la formation réalisée ?** Valeur : en □ minutes □ heures □ jours

**34- Souhaiteriez-vous être sensibilisé(e) aux bonnes pratiques de manipulation des anticancéreux (poster, livret information) en PIPAC ?** □ Oui □ Non

**35- Souhaiteriez-vous être informé(e) aux bonnes pratiques de manipulation des anticancéreux (staff dans le service) en PIPAC ?** □ Oui □ Non

**36- Souhaiteriez-vous être formé(e) aux bonnes pratiques de manipulation des anticancéreux en PIPAC ?**  □ Oui □ Non

**Si oui, durée : □** 1h □ ½ journée □ ou plus

**format : □** E-learning □ Cours magistraux □ Les deux □ Aucun

**37- Selon vous quelles sont les voies de contamination possibles par les anticancéreux en PIPAC?**

1. Voie injectable (piqures) :

*□ D’accord □ plutôt d’accord □ plutôt pas d’accord □ pas d’accord*

1. Voie cutanée :

*□ D’accord □ plutôt d’accord □ plutôt pas d’accord □ pas d’accord*

1. Voie inhalée :

*□ D’accord □ plutôt d’accord □ plutôt pas d’accord □ pas d’accord*

1. Voie oculaire :

*□ D’accord □ plutôt d’accord □ plutôt pas d’accord □ pas d’accord*

1. Voie orale :

*□ D’accord □ plutôt d’accord □ plutôt pas d’accord □ pas d’accord*

1. Autres ?

*□ non □ oui, précisez :*

**38- Selon vous, où peut-on retrouver des anticancéreux au sein du bloc en cas de PIPAC ?**

1. Surface de la poche d’anticancéreux:

*□ D’accord □ plutôt d’accord □ plutôt pas d’accord □ pas d’accord*

1. Surface de l’injecteur de PIPAC:

*□ D’accord □ plutôt d’accord □ plutôt pas d’accord □ pas d’accord*

1. Trocart de PIPAC:

*□ D’accord □ plutôt d’accord □ plutôt pas d’accord □ pas d’accord*

1. Table d’opération:

*□ D’accord □ plutôt d’accord □ plutôt pas d’accord □ pas d’accord*

1. Surface des chariots:

*□ D’accord □ plutôt d’accord □ plutôt pas d’accord □ pas d’accord*

1. Surface des moniteurs:

*□ D’accord □ plutôt d’accord □ plutôt pas d’accord □ pas d’accord*

1. Le respirateur:

*□ D’accord □ plutôt d’accord □ plutôt pas d’accord □ pas d’accord*

1. Téléphone du bloc:

*□ D’accord □ plutôt d’accord □ plutôt pas d’accord □ pas d’accord*

1. Clavier d’ordinateur :

*□ D’accord □ plutôt d’accord □ plutôt pas d’accord □ pas d’accord*

1. Détecteur d’ouverture de porte :

*□ D’accord □ plutôt d’accord □ plutôt pas d’accord □ pas d’accord*

1. Sol :

*□ D’accord □ plutôt d’accord □ plutôt pas d’accord □ pas d’accord*

1. Mur:

*□ D’accord □ plutôt d’accord □ plutôt pas d’accord □ pas d’accord*

1. Aspirateur fumée:

*□ D’accord □ plutôt d’accord □ plutôt pas d’accord □ pas d’accord*

1. Housse du patient :

*□ D’accord □ plutôt d’accord □ plutôt pas d’accord □ pas d’accord*

1. Autres ? :

*□ Non □ Oui, précisez :*

**39- Existe-t-il une procédure spécifique de bionettoyage du bloc en cas de PIPAC ?**

**□** Oui  □ Non □ je ne sais pas

**Si oui, êtes-vous en capacité de l’expliquer ?** □ Oui □ Non

**40- Portez-vous des gants en PIPAC ?**

□ Toujours □ parfois □ jamais

**41- Portez-vous un masque en PIPAC ?**

□ Toujours □ parfois □ jamais

***Si toujours ou parfois,* quel est le type de ce masque ?**  □ FFP1 □ FFP2 □ FFP3

Autres ? : □ Non □ Oui, précisez :

**42- Portez-vous des lunettes de protection en PIPAC ?**

□ Toujours □ parfois □ jamais

**43- Portez-vous une casaque en PIPAC ?**

□ Toujours □ parfois □ jamais

***Si toujours ou parfois,***  **précisez l’équipement :**

**44- En moyenne, combien de PIPACs réalisez-vous par an ?**

□ 0 □ 1-4 □ 5-9 □ 10-14 □ 15-19 □ 20+

**45- Biodécontaminez-vous personnellement les surfaces exposés ? □** Oui  □ Non

**Si oui, a quelle fréquence, biodécontaminez-vous les surfaces exposées ?**

□ 1 fois par changement de patient □ 1 fois par jour

**comment ?**

- Utilisation lingettes (type stericid) **□** Oui  □ Non
- Utilisation de SURFANIOS ou equivalent  **□** Oui  □ Non
- Changez-vous de lingettes à chaque changement de surface ? □ Oui □ Non
- Autre  **□** Non  □ Oui, précisez :

**46- Avez-vous déjà ressenti des troubles (type céphalées) en bloc de PIPAC** **hors exposition accidentelle ?** **□** Oui  □ Non

**47- Avez-vous été exposé(e) à des anticancéreux de façon inhabituelle en PIPAC ?** **□** Oui  □ Non

- **Si non, passez à la partie 4**

**Si oui, à quelle fréquence :**

□ moins d’1fois par an □ 1 fois par an □ une fois par mois □ une fois par semaine □ jamais

**Par quel biais :**

- Déchirures des gants **□** Oui  □ Non
- Au moment de la connexion de la poche à l’injecteur □ Oui □ Non
- Exposition lors de l’élimination DASRI **□** Oui  □ Non
- Autre ?  **□** Non  □ Oui, précisez :

**Suite à ces expositions, avez-vous ressenti des troubles** : **□** Oui  □ Non

- **Si non, passez à la partie 4**

**Qu’avez-vous ressenti ?**

- Signes digestifs **□** Oui  □ Non
- Signes cutanés **□** Oui  □ Non

**Si oui,** - brûlures **□** Oui  □ Non - picotements **□** Oui  □ Non

- Signes neurologiques **□** Oui  □ Non

**Si oui,** - céphalées **□** Oui  □ Non - vertiges **□** Oui  □ Non

- Réactions allergiques **□** Oui  □ Non
- Signes oculaires **□** Oui  □ Non
- Autres ?  **□** Non  □ Oui, précisez :

**De façon générale, si présence d’un trouble quel qu’il soit durée moyenne des symptômes:**

Valeur : en □ minutes □ heures □ jours

**Partie 4 : données personnelles**

**48- Portez-vous des lunettes de vue au travail ? □** Oui  □ Non

**49- Fumez-vous quotidiennement** **?** **□** Oui  □ Non

**50- Avez-vous été traité(e) par des anticancéreux ? □** Oui  □ Non

**Si oui, année du dernier traitement** : **Nom de la** **molécule** :

**51- Dans votre entourage, avez-vous des proches traités par des anticancéreux depuis moins de 3 mois ?** **□** Oui  □ Non

**52- Dans votre entourage, avez-vous des animaux traités par des anticancéreux depuis moins de 3 mois ?** **□** Oui  □ Non
