# Supplementary material for: Perception, knowledge and protective practices for surgical staff handling antineoplastic drugs during HIPEC and PIPAC
Source: Pleura Peritoneum. 2022 Apr 13;7(2):77–86. doi: 10.1515/pp-2021-0151 (PMC9166181; doi:10.1515/pp-2021-0151)
Supplement: Supplementary file 2 — Supplementary Material [file j_pp-2021-0151_suppl_002.docx]

**General questionnaire “CONTACT survey”
HIPEC/PIPAC operating rooms**

**Write in CAPITAL letters, Tick the boxes, Use a black pencil.**

**Surname:**

**First name:**

**Gender: □ Female □ Male**

**Date of birth: |_|_|/|_|_|/|_|_|_|_|**

**Function: □ Surgical nurse (IBODE) □ Nurse anaesthetist (IADE) □ General Nurse (IDE) □ Doctor □ Executive □ Nursing assistant (AS) □ Hospital Nursing Assistant (ASH)**

**□ Intern □ IBODE Student □ IADE Student □ AS/ASH in training**

**□ Other:**

**Are you specifically attached to the operating rooms? □** Yes □ No

**If not, at what hourly rate are you attached to the operating rooms (percentage)?**

**To which other units are you attached?**

**Seniority in the establishment?**  Length: In □ weeks □ months □ years

**Seniority in theatres (non-stop duration)?** Length: In □ weeks □ months □ years

**Part 1: General questions**

**1- Do you consider your overall risk of exposure to antineoplastic drugs to be very low?**

□ Agree □ Disagree □ I don’t know

**2- Between HIPEC and PIPAC, which one presents, according to you, the lesser risk of exposure to antineoplastic drugs?**

□ HIPEC □ PIPAC

**3-Are you scared of handling antineoplastic drugs?**

□ Agree □ Disagree □ I don’t know

**4- Do you think that all antineoplastic drugs present the same risk level of toxicity?**

□ Agree □ Disagree □ I don’t know

**5- Do you think that currently implemented safety measures allow a sufficient reduction of the contamination risk?**

□ Agree □ Disagree □ I don’t know

**6- I am confident in the fact that I can handle every situation where there is a potential exposure to antineoplastic drugs**

□ Agree □ Disagree □ I don’t know

**7- Have you been trained specifically for the handling of antineoplastic drugs during your initial training (nursing school, internship)?** **□** Yes □ No

**8 - Have you been trained specifically for the handling of antineoplastic drugs during your in-house training (in your current establishment)?** □ Yes □ No

**If you have, what is the date of your latest training on the matter?**  |_|_|/|_|_|/|_|_|_|_|

**Duration of the training?** Length: In □ minutes □ hours □ days

**9- Is there a procedure to “follow in case of accidental exposure to antineoplastic drugs”?**

**□** Yes □ No □ I don’t know

**If there is, are you able to detail it?** □ Yes □ No

**10- What do you or would you do in case of exposure to antineoplastic drugs?**

Use of hydroalcoholic gel □ Yes □ No Wash with water □ Yes □ No

Use of soap **□** Yes □ No Nothing **□** Yes □ No

Other?  **□** No □ Yes, please specify:

**11- Do you know what is the procedure for disposing of antineoplastic waste?** □ Yes □ No

**Part 2: Questions on the HIPEC**

**12 - Do you work in a HIPEC operating room?** □ Yes □ No

**If you don’t, skip to part 3.**

**If you do, how long have you been working in HIPEC activity?** Length: In □ weeks □ months □ years

**13**- **How would you assess your risk of exposure to antineoplastic drugs in HIPEC?**

□ Very low □ Low □ High □ Very high

**14- Do you think that the personal protective equipment you are provided is suited (ergonomics/protection) to the handling of antineoplastic drugs in HIPEC?**

□ Agree □ Disagree □ I don’t know

**If you disagree with one, which one is it?**

- Gown(s) **□** Yes □ No - Mask **□** Yes □ No - Protective goggles **□** Yes □ No

- Gloves **□** Yes □ No - Overshoes **□** Yes □ No

- Other? **□** No □ Yes, please specify:

**15- Have you been trained specifically for the handling of antineoplastic drugs in HIPEC during your in-house training (in your current establishment)?** □ Yes □ No

**If you have, what is the date of your latest training on the matter?**  |_|_|/|_|_|/|_|_|_|_|

**Duration of the training?** Length: In □ minutes □ hours □ days

**16- Do you wish to be made aware regarding the handling of antineoplastic drugs (poster, information booklet) in HIPEC?** **□** Yes □ No

**17- Do you wish to be informed regarding the handling of antineoplastic drugs (staff) in HIPEC?** □ Yes □ No

**18- Do you wish to be trained regarding the handling of antineoplastic drugs in HIPEC?**  □ Yes □ No

**If you do, duration: □** 1h □ ½ day □ more

**format: □** E-learning □ Lectures □ Both □ None of the above

**19- What do you think are the potential contamination routes for antineoplastic drugs in HIPEC?**

1. Injectable (via injections)

*□ Agree □ somewhat agree □ somewhat disagree □ disagree*

1. Cutaneous:

*□ Agree □ somewhat agree □ somewhat disagree □ disagree*

1. Inhaled:

*□ Agree □ somewhat agree □ somewhat disagree □ disagree*

1. Ocular:

*□ Agree □ somewhat agree □ somewhat disagree □ disagree*

1. Oral:

*□ Agree □ somewhat agree □ somewhat disagree □ disagree*

1. Other?

*□ No □ Yes, please specify:*

**20- According to you, where can antineoplastic drugs be found in the operating room during HIPEC?**

1. Antineoplastic drugs bag surface:

*□ Agree □ somewhat agree □ somewhat disagree □ disagree*

1. HIPEC pump surface:

*□ Agree □ somewhat agree □ somewhat disagree □ disagree*

1. HIPEC nozzle:

*□ Agree □ somewhat agree □ somewhat disagree □ disagree*

1. Operating table:

*□ Agree □ somewhat agree □ somewhat disagree □ disagree*

1. Trolley surface:

*□ Agree □ somewhat agree □ somewhat disagree □ disagree*

1. Monitor surface:

*□ Agree □ somewhat agree □ somewhat disagree □ disagree*

1. Respirator:

*□ Agree □ somewhat agree □ somewhat disagree □ disagree*

1. Operating room phone:

*□ Agree □ somewhat agree □ somewhat disagree □ disagree*

1. Computer keyboard:

*□ Agree □ somewhat agree □ somewhat disagree □ disagree*

1. Opened door sensor:

*□ Agree □ somewhat agree □ somewhat disagree □ disagree*

1. Floor:

*□ Agree □ somewhat agree □ somewhat disagree □ disagree*

1. Wall:

*□ Agree □ somewhat agree □ somewhat disagree □ disagree*

1. Smoke extractor:

*□ Agree □ somewhat agree □ somewhat disagree □ disagree*

1. Other?

*□ No □ Yes, please specify:*

**21- Is there a specific procedure for biocleaning the operating room during HIPEC?**

□ Yes □ No □ I don’t know

**If there is, are you able to detail it?** □ Yes □ No

**22- Do you wear gloves for HIPEC?**

□ Always □ sometimes □ never

**23- Do you wear a mask for HIPEC?**

□ Always □ sometimes □ never

***If “always” or “sometimes”,* what kind of mask is it?**  □ FFP1 □ FFP2 □ FFP3

Other?: □ No □ Yes, please specify:

**24- Do you wear protective goggles for HIPEC?**

□ Always □ sometimes □ never

**25- Do you wear a gown for HIPEC?**

□ Always □ sometimes □ never

***If “always” or “sometimes”, specify the equipment*:**

**26- On average, how many HIPECs do you carry out yearly?**

□ 0 □ 1-4 □ 5-9 □ 10-14 □ 15-19 □ 20+

**27- Do you personally bio-decontaminate exposed surfaces? □** Yes □ No

**If you do, how often do you bio-decontaminate exposed surfaces?**

□ Once every patient □ Once daily

**How?**

- Use of wipes (stericid type) □ Yes □ No
- Use of SURFANIOS® or equivalent □ Yes □ No
- Do you change wipes for every surface? □ Yes □ No
- Other □ No □ Yes, please specify:

**28- Have you ever felt trouble (like cephalgia) in HIPEC operating room, exclusive of accidental exposure?**

□ Yes □ No

**29- Have you been exposed to antineoplastic drugs in an unusual way?** □ Yes □ No

- **If you haven’t, skip to part 3**

**If you have, how often?**

□ less than once a year □ once a year □ once a month □ once a week □ never

**How:**

- “Overflowing” □ Yes □ No
- Ripped gloves □ Yes □ No
- When connecting the pump to the bag (HIPEC) □ Yes □ No
- When disposing of infectious clinical waste □ Yes □ No
- Other?  □ No □ Yes, please specify:

**Have you experienced trouble following this exposure**: □ Yes □ No

- **If you have not, skip to part 3**

**What have you experienced?**

- Digestive signs □ Yes □ No
- Cutaneous signs □ Yes □ No

**If you have,** - burns **□** Yes □ No - prickling sensation **□** Yes □ No

- Neurological signs □ Yes □ No

**If you have,** - cephalgia **□** Yes □ No - dizziness **□** Yes □ No

- Allergies □ Yes □ No
- Ocular signs □ Yes □ No
- Other?  □ No □ Yes, please specify:

**Generally, if there is any of these, average duration of the symptoms:**

Length: In □ minutes □ hours □ days

**Part 3: Questions on PIPAC**

**30 - Do you work in a PIPAC operating room?** □ Yes □ No

**If you do not, skip to part 4.**

**If you do, how long have you been working in PIPAC activity?** Length: In □ weeks □ months □ years

**31**- **How would you assess your risk of exposure to antineoplastic drugs in PIPAC?**

□ Very low □ Low □ High □ Very high

**32- Do you think that the personal protective equipment you are provided is suited (ergonomics/protection) to the handling of antineoplastic drugs in PIPAC?**

□ Agree □ Disagree □ I don’t know

**If you disagree with one of them, which one is it?**

- Gown(s) **□** Yes □ No - Masks **□** Yes □ No - Protective goggles **□** Yes □ No

- Gloves □ Yes □ No - Overshoes □ Yes □ No

- Other? □ No □ Yes, please specify:

**33- Have you been trained specifically for the handling of antineoplastic drugs in PIPAC during your in-house training (in your current establishment)** □ Yes □ No

**If you have, what is the date of your latest training on the matter?**  |_|_|/|_|_|/|_|_|_|_|

**Duration of the training?** Length: In □ minutes □ hours □ days

**34- Do you wish to be made aware regarding the handling of antineoplastic drugs (poster, information booklet) in PIPAC?** □ Yes □ No

**35- Do you wish to be informed regarding the handling of antineoplastic drugs (staff) in PIPAC? □** Yes □ No

**36- Do you wish to be informed regarding the handling of antineoplastic drugs in PIPAC?**  □ Yes □ No

**If you do, duration: □** 1h □ ½ day □ more

**format: □** E-learning □ Lectures □ Both □ None of the above

**37- What do you think are the potential contamination routes for antineoplastic drugs in PIPAC?**

1. Injectable (via injections)

*□ Agree □ somewhat agree □ somewhat disagree □ disagree*

1. Cutaneous:

*□ Agree □ somewhat agree □ somewhat disagree □ disagree*

1. Inhaled:

*□ Agree □ somewhat agree □ somewhat disagree □ disagree*

1. Ocular:

*□ Agree □ somewhat agree □ somewhat disagree □ disagree*

1. Oral:

*□ Agree □ somewhat agree □ somewhat disagree □ disagree*

1. Other?

*□ No □ Yes, please specify:*

**38- According to you, where can antineoplastic drugs be found in the operating room in the context of PIPAC?**

1. Antineoplastic drugs bag surface:

*□ Agree □ somewhat agree □ somewhat disagree □ disagree*

1. Surface of the PIPAC syringe:

*□ Agree □ somewhat agree □ somewhat disagree □ disagree*

1. PIPAC trocar:

*□ Agree □ somewhat agree □ somewhat disagree □ disagree*

1. Operation table:

*□ Agree □ somewhat agree □ somewhat disagree □ disagree*

1. Trolley surface:

*□ Agree □ somewhat agree □ somewhat disagree □ disagree*

1. Monitor surface:

*□ Agree □ somewhat agree □ somewhat disagree □ disagree*

1. Respirator:

*□ Agree □ somewhat agree □ somewhat disagree □ disagree*

1. Theatre phone:

*□ Agree □ somewhat agree □ somewhat disagree □ disagree*

1. Computer keyboard:

*□ Agree □ somewhat agree □ somewhat disagree □ disagree*

1. Opened door sensor:

*□ Agree □ somewhat agree □ somewhat disagree □ disagree*

1. Floor:

*□ Agree □ somewhat agree □ somewhat disagree □ disagree*

1. Wall:

*□ Agree □ somewhat agree □ somewhat disagree □ disagree*

1. Smoke extractor:

*□ Agree □ somewhat agree □ somewhat disagree □ disagree*

1. Patient’s cover

*□ Agree □ somewhat agree □ somewhat disagree □ disagree*

1. Other? :

*□ No □ Yes, please specify:*

**39- Is there a specific procedure for biocleaning the operating room during PIPAC?**

□ Yes □ No □ I don’t know

**If there is, are you able to detail it?** □ Yes □ No

**40- Do you wear gloves for PIPAC?**

□ Always □ sometimes □ never

**41- Do you wear a mask for PIPAC?**

□ Always □ sometimes □ never

***If “always” or “sometimes”,* what kind of mask is it?**  □ FFP1 □ FFP2 □ FFP3

Other? : □ No □ Yes, please specify:

**42- Do you wear protective goggles for PIPAC?**

□ Always □ sometimes □ never

**43- Do you wear a gown for PIPAC?**

□ Always □ sometimes □ never

***If “always” or “sometimes”, specify the equipment*?**

**44- On average, how many PIPACs do you carry out yearly?**

□ 0 □ 1-4 □ 5-9 □ 10-14 □ 15-19 □ 20+

**45- Do you yourself bio-decontaminate exposed surfaces? □** Yes □ No

**If you do, how often do you bio-decontaminate exposed surfaces?**

□ Once every patient □ Once daily

**How?**

- Use of wipes (stericid type) □ Yes □ No
- Use of SURFANIOS® or equivalent □ Yes □ No
- Do you change wipes for every surface? □ Yes □ No
- Other □ No □ Yes, please specify:

**46- Have you ever felt trouble (like cephalgia) in PIPAC operating room, exclusive of accidental exposure?**

□ Yes □ No

**47- Have you been exposed to antineoplastic drugs in an unusual way in PIPAC?** □ Yes □ No

- **If you have not, skip to part 4**

**If you have, how often?**

□ less than once a year □ once a year □ once a month □ once a week □ never

**How:**

- Ripped gloves □ Yes □ No
- When connecting the bag to the syringe □ Yes □ No
- When disposing of infectious clinical waste □ Yes □ No
- Other?  □ No □ Yes, please specify:

Have you experienced any trouble following this exposure: □ Yes □ No

- **If you haven’t, skip to part 4**

**What have you experienced?**

- Digestive signs □ Yes □ No
- Cutaneous signs □ Yes □ No

**If you have,** - burns **□** Yes □ No - prickling sensation **□** Yes □ No

- Neurological signs □ Yes □ No

**If you have,** - cephalgia □ Yes □ No - dizziness □ Yes □ No

- Allergies □ Yes □ No
- Ocular signs □ Yes □ No
- Other?  □ No □ Yes, please specify:

**Generally, if there is any of these, average duration of the symptoms:**

Length: In □ minutes □ hours □ days

**Part 4: personal data**

**48- Do you wear glasses on the workplace? □** Yes □ No

**49- Do you smoke daily?** □ Yes □ No

**50- Have you been treated with antineoplastic drugs?** □ Yes □ No

**If you have, year of your latest treatment**: **Name of the drug:**

**51- Has one of your relatives been treated with antineoplastic drugs for less than 3 months?**
**□** Yes □ No

**51- Has one of your pets been treated with antineoplastic drugs for less than 3 months?**
**□** Yes □ No
